# Hemostatic Profile and Serum Levels of Interferon Gamma-Induced Protein 10 (IP-10) in Neonates Born to Mothers with COVID-19 During the Peripartum Period

**DOI:** 10.3390/ijms26031201

**Published:** 2025-01-30

**Authors:** Rozeta Sokou, Efstathia-Danai Bikouli, Andreas G. Tsantes, Panagiotis Halvatsiotis, Dimitra Houhoula, Paschalia Taliaka Kopanou, Paraskevi Liakou, Evangelia-Filothei Tavoulari, Daniele Piovani, Stefanos Bonovas, Zoi Iliodromiti, Theodora Boutsikou, Nicoletta Iacovidou, Martha Theodoraki, Argirios E. Tsantes

**Affiliations:** 1 Neonatal Department, Aretaieio Hospital, National and Kapodistrian University of Athens, 11528 Athens, Greece; ziliodromiti@yahoo.gr (Z.I.); theobtsk@gmail.com (T.B.); niciac58@gmail.com (N.I.); 2 Neonatal Intensive Care Unit, “Agios Panteleimon” General Hospital of Nikea, 18454 Piraeus, Greece; danai_mp89@yahoo.gr (E.-D.B.); paschaliakt@gmail.com (P.T.K.); viviliak@gmail.com (P.L.); tavoularieva@yahoo.gr (E.-F.T.); anastasiosmmr@yahoo.gr (M.T.); 3 Microbiology Department, “Saint Savvas” Oncology Hospital, 11522 Athens, Greece; andreas.tsantes@yahoo.com; 4 Second Department of Internal Medicine, Attikon University Hospital, School of Medicine, National and Kapodistrian University of Athens, 12462 Athens, Greece; pahalv@gmail.com; 5 Department of Food Science and Technology, University of West Attica, 12243 Athens, Greece; dhouhoula@uniwa.gr; 6 Department of Biomedical Sciences, Humanitas University, Pieve Emanuele, 20090 Milan, Italy; dpiovani@hotmail.com (D.P.); sbonovas@gmail.com (S.B.); 7 IRCCS Humanitas Research Hospital, 20089 Rozzano, Milan, Italy; 8 Blood Bank Unit, Laboratory of Haematology, “Attiko” Hospital, School of Medicine, National and Kapodistrian University of Athens, 12462 Athens, Greece; atsantes@yahoo.com

**Keywords:** maternal COVID-19, neonatal hemostasis, neonatal immunologic profile, perinatal SARS-CoV-2 exposure, thromboelastometry parameters

## Abstract

The COVID-19 pandemic has raised significant concerns regarding its potential impact on maternal and neonatal health. This study aimed to investigate the immunologic and hemostatic profiles of neonates exposed to SARS-CoV-2 during the peripartum period (0–14 days prior to delivery). This retrospective study included 28 neonates born to COVID-19-positive mothers during the peripartum period and a control group of 54 neonates born to mothers who never tested positive for SARS-CoV-2 during pregnancy. Arterial blood samples were collected from all neonates on the second day of life for the simultaneous assessment of full blood count, C-reactive protein (CRP), serum interleukin-6 (IL-6), and Interferon gamma-induced protein 10 (IP-10) levels, as well as Rotational Thromboelastometry (ROTEM) tests (EXTEM, INTEM, and NATEM). Neonates born to COVID-19-positive mothers and those born to COVID-19-negative mothers exhibited similar coagulation profiles based on ROTEM analysis. Multiple linear regression analysis revealed that peripartum COVID-19 infection was associated with higher IP-10 levels in neonates (coefficient: +16.8, 95% CI: +9.0 to +24.6, *p* < 0.0001). Our study findings suggest that the presence of immunologic disturbance in neonates is related to recent peripartum exposure to maternal SARS-CoV-2 infection, as evidenced by increased IP-10 levels in blood samples obtained from neonates born to SARS-CoV-2-positive mothers. However, peripartum exposure to maternal SARS-CoV-2 did not appear to disrupt the hemostatic profile of the exposed newborns based on ROTEM test results.

## 1. Introduction

The Coronavirus disease 2019 (COVID-19) pandemic is arguably the most impactful infectious disease outbreak of the 21st century. Early detection and prevention of the virus are essential to mitigate its deadly spread. However, given its high transmission potential, predicting the severe disease is crucial for the effective management and optimal allocation of medical resources and personnel. Among the various prognostic strategies being evaluated, biomarker detection has been extensively explored for its potential to predict patient outcomes prior to the onset of severe symptoms. In several inflammatory diseases, excessive cytokine production can trigger a “cytokine storm” immune response. In COVID-19, this phenomenon is characterized by uncontrolled inflammatory responses driven by cytokines such as IP-10. Elevated IP-10 levels induce systemic inflammation, contributing to tissue damage [1] and the development of acute respiratory distress syndrome (ARDS) [2]. Furthermore, elevated IP-10 levels impair T-cell proliferation, leading to increased apoptosis and conditions such as lymphopenia, which correlate directly with a poor prognosis [3]. Although the precise mechanisms involved in the COVID-19 cytokine storm remain incompletely understood, there is a consensus that significantly elevated IP-10 levels result in severe physiological consequences. The targeted inhibition of IP-10 has been proposed as a potential therapeutic strategy to mitigate the cytokine storm. IP-10 has shown significant potential as a diagnostic, prognostic, and therapeutic biomarker of various infectious diseases. In the context of COVID-19, the measurement of IP-10 in serum samples has demonstrated the highest accuracy for both diagnostic and prognostic applications [4].

Pregnant females are known to be markedly susceptible to viral infections, in view of all the physiological and immunological alterations that occur during pregnancy. Maternal immune activation (MIA) is a crucial mechanism involved in the developmental origins of health and disease (DOHaD) [5]. A significant number of pregnant women have been diagnosed as positive for SARS-CoV-2 and the impact on fetuses is variable [6,7,8]. According to available data, the vertical transmission of SARS-CoV-2 may occur at a rate ranging between 1.6% and 6.3%. However, the exact vertical transmission rate has not been assessed so far [9,10,11]. The evaluation of the exact incidence becomes even more challenging because of the variation in the epidemiology among the different strains of SARS-CoV-2 [12,13]. Numerous studies reported an increased rate of preterm labor, the birth of neonates with low birthweight, and an increased rate of neonatal intensive care unit (NICU) admission following maternal infection during pregnancy [14]. Whether the increased rate of NICU admissions can be attributed to morbidity related to the direct effects of SARS-CoV-2 infection or it is caused by the increased incidence of prematurity in this population remains unclear [15,16]. Over the past years, the principles of DOHaD have been implemented in order to investigate the possible impact of SARS-CoV-2 infection, on fetal development in terms of epigenetic alterations with neonatal short- and long-term consequences, such as Respiratory Distress Syndrome (RDS) and neurodevelopmental impairment [6,17]. The effect of intrauterine exposure to SARS-CoV-2 on the neonatal hemopoietic and hemostatic system still remains a dark field under investigation [18].

The aim of our study was to assess the potential effect of neonatal exposure to SARS-CoV-2 in the peripartum period on the hemostatic and immunological profile (as reflected by serum levels of IP-10) of the newborns during a time period that was characterized by the predominance of the *Omicron* strain.

## 2. Results

### 2.1. General Characteristics of Study Groups

The study cohort consisted of 28 neonates, offsprings of SARS-CoV-2-positive mothers during the peripartum period (Group A), and a control group of 54 neonates of SARS-CoV-2-negative mothers during pregnancy (Group B). The demographics of these two groups were similar. Specifically, neonates from both groups had similar gestational age (medians: 38 vs. 38 weeks, *p* = 0.97) and birth weight (medians: 3250 vs. 3300 g, *p* = 0.58). There were 16 (57.1%) males with SARS-CoV-2-positive mothers during the peripartum period and 33 (61.1%) males with SARS-CoV-2-negative mothers during pregnancy (*p* = 0.72). Neonates did not differ between the two groups regarding delivery mode (cesarean delivery: 67.8% vs. 62.9%, *p* = 0.66) or complications during pregnancy. As for the severity of maternal COVID-19 symptoms during pregnancy, 8 (28.6%), 17 (60.7%), and 3 mothers (10.7%) had mild, moderate, and severe symptoms, respectively (based on WHO COVID-19 disease severity categorization [19]). The demographic and clinical parameters of the study population are presented in Table 1.

Only one out of the twenty-eight neonates exposed to SARS-CoV-2 in the peripartum period had positive PCR and remained asymptomatic throughout the observation period. None of the neonates was infected by SARS-CoV-2 during the follow-up and observation period that was extended until the 28th day of life post-discharge as mentioned above.

**Figure 1:**
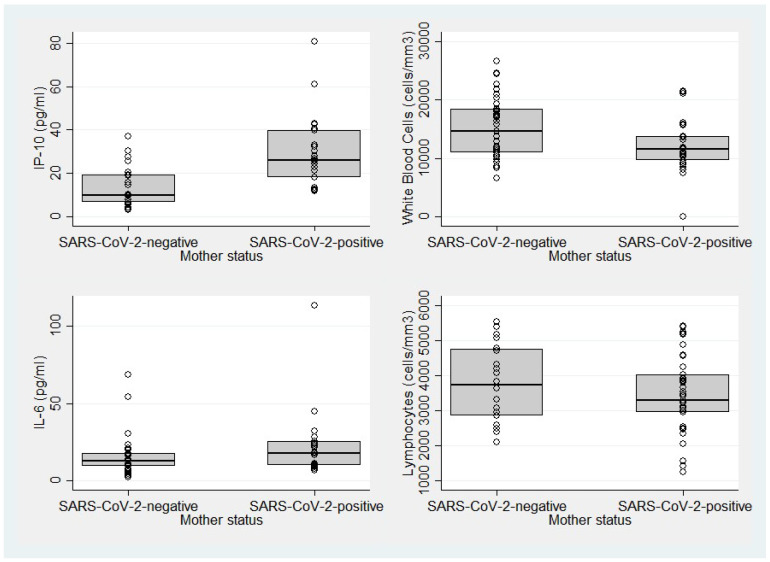
Boxplots of IP-10, IL-6, white blood cell count, and lymphocyte count compared between neonates of SARS-CoV-2-negative and -positive mothers.

Regarding conventional laboratory findings, neonates of both groups had comparable hematocrit levels (medians: 48.0% vs. 48.8%, *p* = 0.73), platelet counts (medians: 293.5 vs. 291.0 × 10^3^/mL, *p* = 0.84), white blood cell counts (medians: 11.5 vs. 14.6 × 10^3^/mL, *p* = 0.023), lymphocytes (medians: 3310 vs. 3740 cells/mm^3^, *p* = 0.44), absolute neutrophil count to absolute lymphocyte count ratio (NLR; 2.8 vs. 1.8, *p* = 0.001), and CRP levels (medians: 1.4 vs. 1.7 mg/L, *p* = 0.46). The laboratory findings of the study population are reported in Table 2 and shown in Figure 1.

### 2.2. ROTEM Parameters and Interleukin Levels

The direct comparison of the ROTEM parameters revealed that neonates in both groups had similar coagulation profiles, as reflected by the ROTEM analysis (Table 3). Specifically, the two groups had similar EXTEM, CT, CFT, A10, MCF, alpha angle, and LI60 values (*p* > 0.05). All the INTEM ROTEM and the NATEM ROTEM parameters were also similar between the two groups (Table 3).

Regarding the cytokine profile, although the IL-6 levels were similar between the two groups (medians: 17.9 vs. 12.6 pg/mL, *p* = 0.17), neonates of SARS-CoV-2-positive mothers had higher IP-10 levels (medians: 26.1 vs. 10.0 pg/mL, *p* < 0.0001; Table 2). IP-10 levels were not correlated with the white blood cell count (*p* = 0.27), the lymphocyte cell count (*p* = 0.98), and the neutrophil count-to-absolute lymphocyte count ratio (*p* = 0.002).

The comparable coagulation profile, as reflected by the ROTEM parameters between neonates of SARS-CoV-2-positive mothers and SARS-CoV-2-negative mothers, was also confirmed by multiple linear regression analysis (adjusted for birth weight, gestational age, gender, delivery mode, and mother’s smoking history; Table 4). Specifically, it was shown that COVID-19 infection during pregnancy was not associated with altered EXTEM, INTEM, and NATEM parameters.

However, multiple linear regression analysis confirmed that COVID-19 infection during pregnancy was associated with an altered cytokine profile of neonates. Specifically, peripartum COVID-19 infection was associated with higher IP-10 levels of the neonates (coefficient: +16.8, 95% CI: +9.0 to +24.6, *p* < 0.0001; Table 4).

## 3. Discussion

The COVID-19 pandemic raised significant concerns regarding its potential impact on maternal and neonatal health and wellbeing. The present study investigated the immunologic (as reflected by serum levels of IP-10) and hemostatic profile of neonates born to mothers with COVID-19 during the peripartum period.

Although the rate of SARS-CoV-2 PCR positive tests in the neonates of our study population was nearly zero (except for one neonate), and therefore, there was limited vertical transmission of SARS-CoV-2, the data derived from our study suggest immunological disturbances in this population, related to the recent peripartum exposure to maternal COVID-19, as reflected by the increased concentrations of IP10 in the blood samples obtained from neonates of mothers SARS-CoV-2-positive mothers, compared to the neonates from mothers who were not exposed to the virus. These findings, therefore, confirm available published data that indicate the presence of an immunological inheritance that the neonate acquires following perinatal exposure to SARS-CoV-2, with potentially significant consequences both in the short- and long-term. The hematological parameters that were investigated in the neonates exposed to COVID-19 were comparable between the study groups and were all within the normal range for the respective age group and day of life; this finding was in line with previous studies [18,20,21]. None of the hematological disturbances that are usually evident in COVID-19 infection in adults was traced, and placental insufficiency in the fetus was not noted either [22,23,24]. Furthermore, peripartum maternal COVID-19 infection did not lead to disturbances of the hemostatic profile of the newborns who were exposed, according to the data from the ROTEM tests assessment.

Intrauterine inflammation is known to be a cause of unfavorable pregnancy outcomes, including preterm onset of labor and intrauterine fetal demise [25,26]. There are indications that pregnant women who are infected with SARS-CoV-2 have a systemic inflammatory response that is characterized by increased concentrations of cytokines, such as the interleukins IL-8, IL-10, and IL-15 in the systematic circulation [18,26]. Increased levels of proinflammatory cytokines such as IL-6, IL-8, and IP-10 were also reported in the blood of neonates born to these mothers thus suggesting the existence of perinatal systemic inflammation [18,26]. There is significant evidence that SARS-CoV-2 infection not only increases maternal cytokine levels but also triggers immune alterations in the fetus, linked to MIA rather than direct fetal contamination [26].

The role and involvement of IL-6 in the pathogenesis of COVID-19 disease have been thoroughly studied. In COVID-19 patients, IL-6 levels are significantly elevated and related to unfavorable clinical outcomes [27]. Several studies reported significantly higher levels of serum IL-6 both in maternal and fetal blood following exposure to SARS-CoV-2 in comparison with healthy controls [28,29,30,31]. Contrarily, our study showed that peripartum COVID-19 infection is not associated with altered IL-6 levels. Although neonates born to affected mothers exhibited slightly higher IL-6 levels, this difference was not statistically significant. This discrepancy with the above-mentioned studies could be attributed to the fact that, in our study, we included neonates who were “apparently” healthy, meaning that they did not have any of the direct complications related to intrauterine exposure to SARS-CoV-2. Moreover, the majority of the mothers who were included in our study had mild-to-moderate symptoms and none of them was severely ill. IL-6 levels in maternal and neonatal blood are known to be related to infection severity [31,32]. Last but not least, a significant factor that could also contribute to the different results of our study, with regard to the IL-6 levels, is the time of the sample collection, which was between the 2nd and 3rd day of life in our study, as opposed to umbilical cord samples that were obtained in other studies. Moreover, data from the literature suggest that the presence of elevated IL-6 levels in neonates with early onset sepsis is related to blood sampling within the first 24 h of life [33].

Studies suggested that the levels of IP-10 are increased at an earlier stage compared to other proinflammatory cytokines in patients with COVID-19 and that they are closely related to the disease course. Moreover, in adult patients with COVID-19, IP-10 has a long-term profile of persistently increased serum levels that is distinct from its secretion pattern in other viral infections [34]. In our study, multiple linear regression analysis confirmed that COVID-19 infection during the peripartum period is associated with higher IP-10 levels in the neonates. These findings were in accordance with other studies that suggested the presence of elevated IP-10 levels in maternal, and more significantly, neonatal blood serum following maternal COVID-19 infection during pregnancy [28,29]. During pregnancy, elevated maternal IP-10 levels were associated with miscarriages and preeclampsia [35,36]. In addition to this, IP-10 was characterized as one of the chemokines with the strongest angiostatic activity, and can potentially contribute to lung injury and lead to alteration of pulmonary vascular and alveolar development in preterm babies that are exposed to maternal chorioamnionitis [37]. Preliminary results from several studies underline the potential correlation between prenatal exposure to SARS-CoV-2 and the poorer development of the motor skills and interactive behavior of infants [38,39,40]. In addition to this, an elevated risk of respiratory distress syndrome was reported in term neonates that had been exposed to, but not infected by, SARS-CoV-2 [17]. The increased levels of maternal inflammatory cytokines, as a result of infection during pregnancy, were reported to potentially disrupt several characteristics of the fetal brain and lung development [41,42]. The extent to which elevated IP-10 levels in the neonates exposed to SARS-CoV-2 during pregnancy can contribute to the respiratory and neurodevelopmental unfavorable outcome of this population remains to be further investigated.

Inflammation and coagulation are two pathophysiological processes closely intertwined that significantly affect one another in a two-way manner. The activation of the coagulation mechanism constitutes a significant part of the human response to all kinds of inflammatory conditions and subsequently to its immunological defense [43,44,45,46]. At the same time, systemic inflammation constitutes a strong prothrombotic response, since the activated immunological mechanisms induce the production of procoagulant factors, inhibit the natural anticoagulant factors and fibrinolytic activity, and last but not least, increase platelet reactivity [47]. The presence of increased levels of proinflammatory interleukins was shown in vitro to alter the qualities of clot firmness, as assessed with viscoelastic methods [46]. The hemostatic profile of COVID-19 patients was studied by Mitrovic et al. [48], who reported the presence of a hypercoagulant state, expressed through clot formation acceleration, increased maximum clot firmness, and reduced fibrinolysis in ROTEM analysis, as the most common finding in patients with severe disease and increased levels of IL-6.

In our study, all the examined ROTEM parameters, in line with IL-6 levels, were similar between the groups, and there were no indications of hypercoagulability in the COVID-19 group. This finding is in accordance with a previous study, in which the authors concluded that maternal COVID-19 infection did not lead to hypercoagulability in fetal circulation when assessed with Calibrated Automated Thrombography in umbilical cord blood samples [18]. Although COVID-19 infection may cause hypercoagulability in adult patients [49] and thrombosis in fetal and maternal chorionic villi [50], it does not appear to lead to hypercoagulability in the fetus. However, there was neither hypercoagulability nor any other hemostatic disturbance in the group of exposed neonates to SARS-CoV-2 in the peripartum period. The absence of hemostatic derangement in this group of neonates is a discovery, suggesting that the fetus is somehow protected from the maternal systemic infection and inflammatory response. An additional factor that could explain the absence of hemostatic derangement in this group of neonates is the time period of the study conducted—from January to December 2022—when the Omicron variant was dominant. The fact that the ROTEM parameters did not show significant differences is not surprising, as the Omicron variant is not thrombogenic, unlike other types of the virus [51]. Furthermore, results from studies in adults indicate that patients with COVID-19 exhibit different characteristics in blood tests and coagulation status, depending on the infecting variant. Specifically, coagulation disorders are less severe in patients infected with the Omicron variant compared to those infected with the Wild Type, Alpha, or Delta strains [52,53].

To our knowledge, this is the first study aiming to assess the hemostatic profile of newborns following maternal SARS-CoV-2 diagnosis during the peripartum period. For the evaluation of the hemostatic status of the neonates, we utilized the method ROTEM, which is a global coagulation assay, since the conventional clotting tests, despite providing significant information regarding the activation of the blood clotting cascade and the consumption of the clotting factors, have a doubtful diagnostic efficacy as well as limitations in the prediction of significant bleeding and in the guidance of transfusion therapy administration in critically ill patients and especially neonates [54,55,56]. In addition to this, in neonates, who are a vulnerable and sensitive age group where laboratory testing is difficult to perform and interpret, thromboelastography begins to find its role in the neonatologists’ diagnostic options and capabilities [57,58,59,60,61,62,63].

The present study has limitations. Our sample population was small and consisted of a larger percentage of neonates with maternal COVID-19 infection with mild or moderate symptoms, while the neonates themselves were asymptomatic and apparently healthy. Due to its small sample size, our data set may have a highly variable distribution; thus, our results should be interpreted with caution. Also, given the large number of statistical comparisons, the significance threshold was adjusted using the Bonferroni correction to control for multiple testing. However, this approach is considered conservative, especially in observational studies with a small sample size, a large number of tests, and when tests are not independent. Another limitation of this study is that it involves neonates exposed to SARS-CoV-2 in the peripartum period but does not provide information regarding the effect of a potential exposure to SARS-CoV-2 in different previous stages of gestation. A consecutive analysis and larger cohorts are needed in order to fully depict and characterize the immunologic and hemostatic profile of the exposed neonates, especially in relation to gestational age during the maternal infection and the severity of the latter. In addition to these, given the high incidence of asymptomatic COVID-19 infections (that may remain undiagnosed), it is possible that in the control group, women who were exposed to SARS-CoV-2 during gestation and remained undiagnosed may have been included. However, this asymptomatic infection (if it occurred) is not very likely to have affected the results of our study. It is worth noting, though, that due to strict selection criteria of the study population prior to recruitment and based on the protocol of the management of pregnant women in our hospital during the pandemic, none of the women that were included in the control group had COVID-19 disease in the peripartum period. Finally, the fact that we did not obtain blood samples from the mothers at the same time with their neonates for measurement of interleukin levels, as well as the absence of placental tissue examination made the potential of comparison unfeasible, and this could also be considered as a limitation of the study.

## 4. Materials and Methods

The study population of this retrospective study consisted of neonates delivered at the Maternity Clinic of the General Hospital of Nikea-Piraeus “Agios Panteleimon” between January and December 2022 and were allocated in two groups. Group A included neonates with confirmed perinatal exposure to SARS-CoV-2 from the time point 0 to 14 days prior to delivery (newborns whose mothers manifested with symptoms consistent with COVID-19 and tested positive for SARS-CoV-2 RNA in polymerase chain reaction (PCR) testing performed in a reference laboratory).

Group B included neonates with no history of perinatal exposure to SARS-CoV-2 (newborns whose mothers tested negative for SARS-CoV-2 RNA (PCR) during the time period of 0–14 days prior to delivery, and in addition, they never tested positive in either PCR or rapid antigen tests performed during pregnancy and never presented with symptoms indicative of COVID-19 infection during pregnancy).

### 4.1. Study Exclusion Criteria

Neonate offsprings of mothers with factors predisposing to neonatal sepsis such as chorioamnionitis, GBS colonization, delivery before 37 weeks, and rupture of membranes prolonged more than 18 h or with suspected congenital infection were excluded from the study. Furthermore, neonates whose mothers had preeclampsia and/or gestational diabetes and positive personal or family history of coagulopathy were also excluded.

### 4.2. Hospital Policy for the Management of Pregnant Women and Neonates During the COVID-19 Pandemic

All pregnant women who were booked for a scheduled obstetric assessment and examination had to have a PCR test for SARS-CoV-2. If the test was positive, they remained at the Maternity ward in a specified area for COVID-19-positive pregnant women. If the birth was imminent, either vaginal or via caesarian section, the woman was transferred to a delivery suite or operation theater that had been prepared for these cases with the indicated equipment, as per international guidelines, in order to avoid viral transmission.

With regard to the asymptomatic term/late preterm neonates, who mainly constituted our study population, rooming in with their mothers and exclusively breastfeeding are known to be the optimal way of care during their stay at the nursery. Therefore, in the cases of confirmed maternal COVID-19 infection, either asymptomatic or with mild disease, the asymptomatic newborn was hospitalized in the same room with their mother, at least 2 m away from her, and personal protective equipment (PPE; mask and hand hygiene) was applied constantly. In the cases of rooming-in, written consent was obtained from the mother.

The newborn was tested with SARS-CoV-2 PCR (nasopharyngeal swab) at 24 h of life, and subsequently at 48–72 h of life. If the clinical condition of the neonate was not compromised, they were discharged home, and the carers were instructed regarding caring at home with protection measures until the mother remained afebrile for at least 3 days free of antifebrile medication, and until more than 10 days had elapsed from the time of onset of symptoms. The management of neonates included the close monitoring of the neonate’s clinical course by regular phone communication. Follow-ups and observations were extended post-discharge until the 28th day of life.

### 4.3. Records and Laboratory Testing

Family history, obstetric, and perinatal and neonatal history, as well as demographics, were recorded including gender, birthweight, gestational age, type of pregnancy (singleton, twin, or multiple), type of labor (vaginal delivery or caesarian section), presence of small for gestational age (SGA; defined as a newborn with a birthweight below the 10th centile for gestational age) [64], presence of intrauterine growth restriction (IUGR; defined as a fetus that fails to attain their growth potential during intrauterine growth and development) [64].

An arterial blood sample of 2.5 mL was drawn using a 21 G needle on the 2nd day of life for full blood count and biochemistry, serum IL-6 and IP-10 levels, and thromboelastometry; 1 mL was immediately transferred into a 1 mL sodium citrate tube (9NC, 0.109 mol/L, 3.2%), and 300 μL of this sample were used for each ROTEM^®^ assay. Biochemistry analysis (transaminases, urea, creatinine, bilirubin, glucose, calcium, electrolytes, etc.) was performed using the EXL DIMENSION Analyzer (SIEMENS, Healthcare Diagnostics, Newark, DE, USA). C-reactive protein (CRP) level evaluation was performed using the Particle-Enhanced Turbidimetric Immunoassay Technique (PETIA). With regard to the thromboelastometry parameter assessment, we utilized the Rotational Thromboelastometry device ROTEM. The ROTEM tests that were performed consisted of the following:

ΕΧΤΕΜ: An assessment of the activity of the extrinsic pathway of the coagulation cascade (clotting factors VII, X, V, and II and fibrinogen) with the addition of calcium and the activator of the extrinsic pathway of the coagulation cascade (thromboplastin-TF).

INTEM: An assessment of the intrinsic pathway of the coagulation cascade (clotting factors XII, XI, IX, VIII, X, V, and II and fibrinogen) using the addition of calcium and the activator of the intrinsic pathway of the coagulation cascade (ellagic acid).

ΝΑΤΕΜ: It is performed with only the addition of calcium to the blood sample and the clot is expected to form without the addition of other reagents—activators. This test is particularly sensitive to intrinsic activators, such as the expression of tissue factors in cases of inflammation, sepsis, and liver cirrhosis.

The ROTEM parameters that were assessed were the following: clotting time (CT, in seconds), clot formation time (CFT, in seconds), clot strength value in time of 10 min from CT (A10, mm), α-angle of clot kinetics (a°), maximum clot firmness (MCF, in mm), and the lysis index 60 min after clotting time (LI60, %, respectively).

All neonates that were included in the study received 1 mg of Vitamin K intramuscularly immediately after birth as per guidelines.

### 4.4. Determination of IL-6 and Human IP-10 (CXCL10)

Blood samples were drawn into tubes without anticoagulant, and were allowed to clot for 30 min. Then, tubes were centrifuged at 2000× *g* for 10 min at 4 °C and aliquots of samples were stored at −40 °C for measurement. Serum samples were transported to the laboratory within a week after collection.

**Figure 2:**
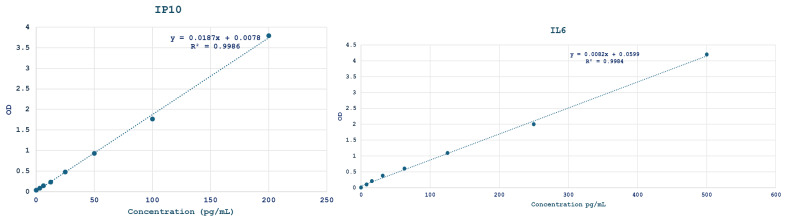
Standard curve of Human IL-6 and IP-10.

Serum levels of IL-6 and Human IP-10 were measured using commercial ELISA kits from Abcam, Cambridge, UK (catalog number for IL-6ab178013: and for Human IP-10: ab173194) as per the manufacturers’ protocols. Samples, including standards of known Human IP-10 Lyophilized Recombinant Protein and Human IL-6 Lyophilized Recombinant Protein content, and samples of the protocol of our study, were pipetted into these wells. During the first incubation, Human IP-10 and IL-6 antigens bound to the immobilized (capture) antibodies on one site. After washing, biotinylated monoclonal detector antibodies specific to Human IP-10 and IL-6 were added. During the second incubation, this antibody bound to the immobilized Human IP-10 and IL-6 captured during the first incubation. After the second incubation, a substrate solution was added, which was acted upon by the bound enzyme to produce color. We measured the absorbance of each well at 450 nm, after adding 100 μL of stop solution. The intensity of this colored product was directly proportional to the concentration of Human IP-10 and IL-6 present in the original specimen. All the standards, controls and samples were measured in triplicates. The mean absorbance was calculated for each set. A linear regression analysis (r^2^ value was 0.99) was used to generate a standard curve. The concentrations of the samples were calculated according to the linear equations (Figure 2).

#### 4.4.1. Assay Sensitivity

The sensitivity and minimum detectable dose of Human IP-10 and IL-6 using this Abcam ELISA kit was 2.6 and 2 pg/mL, respectively. 

#### 4.4.2. Precision

The precision of the Intra and Inter assay was calculated in 6 samples measuring the percentage of coefficient of variation (CV%). In the control group (healthy term neonates of the nursery), the respective laboratory testing was performed on the 2nd–3rd day of life, on the occasion of blood sampling for any medical reason (i.e., investigation of neonatal jaundice).

### 4.5. Statistical Analysis

Descriptive statistics were calculated for demographics, laboratory findings, and clinical parameters, comparing these parameters between neonates born to SARS-CoV-2-positive mothers during the peripartum period and neonates born to SARS-CoV-2-negative mothers for the virus. The normality of data distribution was evaluated using the Shapiro-Wilk test, indicating the deviation of our data set from a normal distribution. Therefore, comparisons were made using the chi-square test for categorical variables and the non-parametric Wilcoxon rank-sum test for continuous variables. Moreover, a correlation between laboratory parameters was evaluated using Spearman’s rank correlation test.

To assess whether peripartum COVID-19 infection independently influenced neonatal coagulation and cytokine profiles, as indicated by ROTEM and interleukin parameters, multiple linear regression analysis was conducted. Associations were adjusted for potential confounders, including gestational age, birth weight, gender, delivery mode (cesarean vs. vaginal), and maternal smoking history. Each multiple linear regression model included one laboratory parameter (of ROTEM parameters and interleukins) as a dependent variable, and gestational age, birth weight, gender, delivery mode, and maternal smoking history as independent variables.

Statistical analysis was performed using Stata 15.0 software (Stata Corp., College Station, TX, USA). Initially, statistical significance was set at *p* < 0.05 for all tests. However, given the large number of statistical comparisons, the significance threshold was adjusted using the Bonferroni correction to control for multiple testing. Consequently, statistical significance was redefined as *p* < 0.0008 for all analyses.

## 5. Conclusions

This study supports an increasing body of evidence in the literature which suggests that perinatal alterations from maternal COVID-19 infection during pregnancy may affect neonatal health and wellbeing, even in the absence of fetal transmission of SARS-CoV-2. Indeed, according to our study findings, exposure of the fetus to the virus prior to delivery appears to be related to immunological disruptions in the newborn, as reflected by the elevated levels of IP-10. Furthermore, this study provides some data and reassurance regarding the hematological results of infants exposed to COVID-19 in utero in the peripartum period. No indications of hypercoagulability or other hemostatic disturbances of the exposed neonates in the peripartum period were highlighted. Future studies that will include larger patient groups, as well as preclinical models, will be necessary in order to characterize and define the pathogenic mechanisms and developmental impact of the peripartum exposure to SARS-CoV-2 on the immunological and hemostatic profile of the neonates that are determined in this study. Finally, the present study underlines the importance of the long-term follow-up of all neonates, born to mothers who were infected with SARS-CoV-2 during pregnancy, including not only asymptomatic ones but also SARS-CoV-2-exposed uninfected ones as they can be considered to be a high-risk population of the COVID-19 era.

## Figures and Tables

**Table 1:** Characteristics of the study population.

	Newborns of SARS-CoV-2-Positive Mothers (*n* = 28)	Control Group (*n* = 54)	*p*-Value
Gestational age (weeks)	38.0 (38.0–39.0)	38.0 (38.0–39.0)	0.97
Gender (males)	16 (57.1)	33 (61.1)	0.72
Birth weight (g)	3255 (3030–3560)	3300 (3070–3525)	0.58
Delivery mode	19 (67.8)	34 (62.9)	0.66
Preterm	2 (7.1)	2 (3.7)	0.49
Problems and complications during pregnancy			
Intrauterine growth restriction	1 (3.5)	0 (0.0)	0.16
Smoking during pregnancy	4 (14.2)	5 (9.2)	0.49
COVID-19 compatible symptoms			
Asymptomatic	0 (0.0)	54 (100)	N/A
Mild	8 (28.6)	0 (0.0)	
Moderate	17 (60.7)	0 (0.0)	
Severe	3 (10.7)	0 (0.0)	

Data are presented as medians and interquartile range (IQR) or as frequencies (percentages) when appropriate.

**Table 2:** Conventional laboratory studies of the study groups.

Variables	Newborns of SARS-CoV-2-Positive Mothers (*n* = 28)	Control Group (*n* = 54)	*p*-Value
WBC (cells/mm^3^)	11,500 (9700–13,800)	14,600 (11,000–18,400)	0.023
Lymphocytes (cells/mm^3^)	3310 (2950–4020)	3740 (2860–4755)	0.44
NLR	2.8 (1.9–3.6)	1.8 (1.1–2.5)	0.001
Lymphocytes (%)	23.0 (19.0–29.2)	27.6 (24.2–36.5)	0.003
Hct (%)	48.0 (45.1–50.1)	48.8 (44.7–51.6)	0.73
PLTs (count × 10^3^/mL)	293.5 (241.5–360.5)	291.0 (242.0–336.0)	0.84
CRP (mg/L)	1.4 (1.0–2.3)	1.7 (1.1–4.1)	0.46
SGOT (IU/L)	51.0 (41.0–65.0)	60.0 (45.5–75.5)	0.10
SGPT (IU/L)	21.0 (14.0–35.0)	24.0 (20.0–34.0)	0.36
HsTI (ng/L)	169.9 (48.9–291.0)	29.8 (18.8–45.7)	0.20
CK (IU/L)	281.0 (188.0–480.0)	667.0 (408.0–941.0)	0.13
TBIL (mg/dL)	6.9 (4.6–11.2)	9.4 (6.6–11.0)	0.15
DBIL (mg/dL)	0.3 (0.2–0.3)	0.2 (0.2–0.3)	0.69
IL-6 (pg/mL)	17.9 (10.1–25.7)	12.6 (9.6–17.7)	0.17
IP-10 (pg/mL)	26.1 (18.3–39.7)	10.0 (6.5–19.2)	**<0.0001**
Blood group			0.66
O	7 (35.0)	12 (26.0)	
A	8 (40.0)	23 (50.0)	
B	3 (15.0)	9 (19.5)	
AB	2 (10.0)	2 (4.3)	

Abbreviations: WBC, white blood cells; NLR, absolute neutrophil count to absolute lymphocyte count ratio; PLTs, platelets; CRP, C-reactive protein; CK, creatine kinase; Hct, hematocrit test; HsTI, high-sensitivity cardiac troponin I; TBIL, total bilirubin; DBIL, direct bilirubin; SGOT, serum glutamic-oxaloacetic transaminase; SGPT, serum glutamic pyruvic transaminase; IP-10, interferon-inducible protein-10; IL-6, Interleukin 6. Data are presented as medians and interquartile range (IQR) or as frequencies (percentages) when appropriate.

**Table 3:** ROΤΕΜ parameters among the study groups.

Variables	Newborns of SARS-CoV-2-Positive Mothers (*n* = 28)	Control Group (*n* = 54)	*p*-Value
EXTEM CT (s)	59.0 (53.5–76.0)	67.5 (58.0–81.0)	0.14
EXTEM CFT (s)	93.0 (76.5–125.0)	90.0 (72.0–113.0)	0.60
EXTEM A10 (mm)	52.0 (46.5–56.0)	52.0 (46.0–56.0)	0.73
EXTEM MCF (mm)	58.5 (55.5–62.5)	59.0 (55.0–61.0)	0.81
EXTEM Alpha angle (°)	71.5 (67.0–75.5)	72.0 (69.0–75.0)	0.96
EXTEM LI60 (%)	95.0 (94.0–97.0)	96.0 (93.0–97.0)	0.75
INTEM CT (s)	218.5 (205.0–242.0)	216.5 (199.0–233.0)	0.61
INTEM CFT (s)	81.0 (66.0–88.0)	83.0 (69.0–101.0)	0.51
INTEM A10 (mm)	54.5 (51.0–57.0)	53.0 (50.0–56.0)	0.47
INTEM MCF (mm)	59.5 (57.0–63.0)	60.0 (56.5–63.0)	0.98
INTEM Alpha angle (°)	74.0 (72.0–78.0)	74.0 (71.0–76.5)	0.35
INTEM LI60 (%)	94.0 (92.0–96.0)	95.0 (93.0–97.0)	0.62
NATEM CT (s)	489.5 (376.0–563.0)	436.0 (317.0–574.0)	0.59
NATEM CFT (s)	142.0 (112.0–177.0)	140.5 (105.5–173.5)	0.74
NATEM A10 (mm)	48.0 (41.0–54.0)	47.0 (43.0–52.0)	0.64
NATEM MCF (mm)	58.0 (51.0–60.0)	58.0 (54.0–61.0)	0.72
NATEM Alpha angle (°)	63.5 (57.0–68.0)	63.0 (58.0–69.0)	0.91
NATEM LI60 (%)	94.0 (93.0–96.0)	95.0 (94.0–97.0)	0.23

Abbreviations: CT, clotting time; CFT, clot formation time; A10, clot amplitude at 10 min; MCF, maximum clot firmness; LI60, lysis index at 60 min; Data are presented as medians and interquartile range (IQR).

**Table 4:** Results of multivariable linear regression analyses for ROTEM parameters, IL-6, and IP-10 as dependent variables, with gestational age, birth weight, gender, delivery mode (cesarean vs. vaginal), mothers smoking history, and mothers COVID-19 status (positive vs. negative) as independent variables.

Variables	SARS-CoV-2-Exposure		
	Coefficient	95% CI	*p*-Value
EXTEM CT (s)	−18.0	−50.2 to +14.1	0.26
EXTEM CFT (s)	−7.2	−51.4 to +36.8	0.74
EXTEM A10 (mm)	+1.3	−3.5 to +6.1	0.58
EXTEM MCF (mm)	+1.5	−3.0 to +6.1	0.51
EXTEM alpha angle (°)	+24.5	−62.8 to +111.9	0.57
EXTEM LI60 (%)	+1.2	−0.8 to +3.3	0.24
INTEM CT (s)	+4.0	−13.4 to +21.4	0.64
INTEM CFT (s)	−6.7	−22.9 to +9.4	0.40
INTEM A10 (mm)	+2.1	−2.4 to +6.6	0.35
INTEM MCF (mm)	+0.9	−3.5 to +5.5	0.66
INTEM alpha angle (°)	+2.7	−2.5 to +8.0	0.30
INTEM LI60 (%)	−0.1	−1.6 to +1.4	0.87
NATEM CT (s)	−1.8	−82.2 to +79.5	0.96
NATEM CFT (s)	+3.2	−29.4 to +35.9	0.84
NATEM A10 (mm)	+1.2	−3.1 to +5.5	0.57
NATEM MCF (mm)	+0.4	−3.7 to +4.6	0.81
NATEM Alpha angle (°)	−0.7	−4.8 to +3.4	0.72
NATEM LI60 (%)	−0.5	−2.1 to +1.0	0.49
IL-6 (pg/Ml)	+4.9	−6.0 to +15.9	0.36
IP-10 (pg/Ml)	+16.8	+9.0 to +24.6	**<0.0001**

Abbreviations: CI, confidence interval; CT, clotting time; CFT, clot formation time; A10, clot amplitude at 10 min; MCF, maximum clot firmness; LI30, lysis index at 30 min; LI60, lysis index at 60 min; IP-10, interferon-inducible protein-10; IL-6, Interleukin 6.

## Data Availability

The original contributions presented in the study are included in the article, further inquiries can be directed to the corresponding author.
